# P-1458. The Implementation Science Coordination Initiative (ISCI) for evidence-based HIV services: supporting implementation in practice

**DOI:** 10.1093/ofid/ofae631.1630

**Published:** 2025-01-29

**Authors:** Virginia McKay, Alithia Zamantakis, Ana Pachicano, James Merle, Morgan Purrier, Russel Brewer, Dennis Li, Justin Smith, Nanette Benbow, Brian Mustanski

**Affiliations:** Washington University in St. Louis, Saint Louis, MO; Northwestern University, Chicago, Illinois; Northwestern University, Chicago, Illinois; University of Utah, Salt Lake City, Utah; Northwestern University, Chicago, Illinois; University of Chicago, Chicago, Illinois; Northwestern University, Chicago, Illinois; University of Utah, Salt Lake City, Utah; Northwestern University, Chicago, Illinois; Northwestern University, Chicago, Illinois

## Abstract

**Background:**

The Ending the HIV Epidemic (EHE) Initiative was launched in 2019 to address implementation problems related to HIV interventions. In alignment with EHE, the National Institutes of Health supported research projects focusing on implementation science (IS), or the scientific examination of strategies to overcome barriers to and improve effective implementation of HIV-related interventions. The result is a growing IS knowledge base. The Implementation Science Coordination Initiative (ISCI) at Northwestern University has developed a suite of tools and resources for providers for practice communities to improve implementation and uptake of HIV prevention, testing, and treatment using IS.

**Figure:**
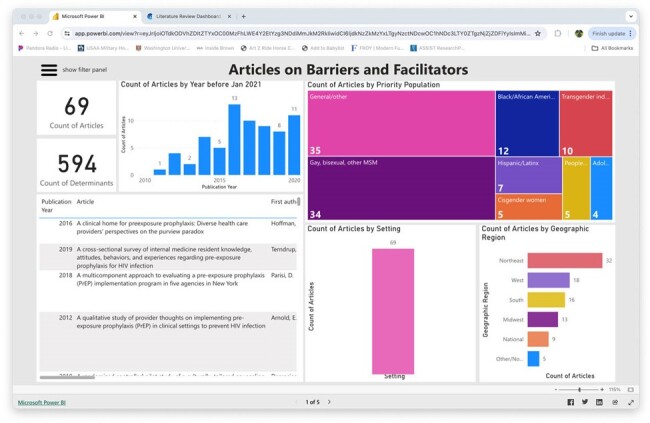

**Methods:**

We will describe tools, projects, and programs available to clinicians to support implementation of evidence-based HIV interventions in practice including that have been developed over from 2020-2024.

**Results:**

- ISCI maintains a literature review dashboard for that summarizes ongoing systematic reviews of the HIV implementation science literature. It includes common barriers and facilitators to evidence-based HIV interventions, interventions to support patients, and implementation strategies for practitioners and clinics. Literature is filterable along various dimensions so that users can quickly review literature relevant to their setting and patient population. See Figure 1.
- ISCI provides two training opportunities for practitioners interested in learning about IS and innovative tools that can be applied to real-world practice. The IS Navigation Practitioner Page is an online, freely available self-paced curriculum. The Practitioner-Oriented Implementation Science Education (POISE) Training Program is a mix of in-person and remote training over a 5-month period leveraging IS to support implementation in one’s practice setting.
- The Best Practices Project identifies and disseminates implementation strategies for evidence-based HIV interventions with the best evidence. We accomplish this through a review process and then develop a mix of resources to support use of implementation strategies in practice, which are freely accessible on the ISCI webpage

**Conclusion:**

There are a number of resources available to support evidence-based HIV services.

**Disclosures:**

**Brian Mustanski, PhD**, Hologic: Grant/Research Support

